# Dissecting indirect genetic effects from peers in laboratory mice

**DOI:** 10.1186/s13059-021-02415-x

**Published:** 2021-07-26

**Authors:** Amelie Baud, Francesco Paolo Casale, Amanda M. Barkley-Levenson, Nilgoun Farhadi, Charlotte Montillot, Binnaz Yalcin, Jerome Nicod, Abraham A. Palmer, Oliver Stegle

**Affiliations:** 1 European Molecular Biology Laboratory, European Bioinformatics Institute, Wellcome Genome Campus, CB10 1SD Hinxton, Cambridge, UK; 2 Department of Psychiatry, University of California San Diego, La Jolla, CA 92093 USA; 3 Current Address: Centre for Genomic Regulation (CRG), The Barcelona Institute of Science and Technology, Dr. Aiguader 88, 08003 Barcelona, Spain; 4 Microsoft Research New England, Cambridge, MA USA; 5 INSERM U1231 GAD Laboratory, University Bourgogne Franche-Comté, 21070 Dijon, France; 6 Wellcome Centre for Human Genetics, University of Oxford, Oxford, UK; 7 Current Address: The Francis Crick Institute, London, UK; 8 Institute for Genomic Medicine, University of California San Diego, La Jolla, CA 92093 USA; 9 European Molecular Biology Laboratory, Genome Biology Unit, Heidelberg, Germany; 10 Division of Computational Genomics and Systems Genetics, German Cancer Research Center, 69120 Heidelberg, Germany; 11 Wellcome Sanger Institute, Wellcome Genome Campus, CB10 1SD Hinxton, Cambridge, UK

**Keywords:** Indirect genetic effects, Social genetic effects, Peer effects, Genotype to phenotype, Genome-wide association study

## Abstract

**Background:**

The phenotype of an individual can be affected not only by the individual’s own genotypes, known as direct genetic effects (DGE), but also by genotypes of interacting partners, indirect genetic effects (IGE). IGE have been detected using polygenic models in multiple species, including laboratory mice and humans. However, the underlying mechanisms remain largely unknown. Genome-wide association studies of IGE (igeGWAS) can point to IGE genes, but have not yet been applied to non-familial IGE arising from “peers” and affecting biomedical phenotypes. In addition, the extent to which igeGWAS will identify loci not identified by dgeGWAS remains an open question. Finally, findings from igeGWAS have not been confirmed by experimental manipulation.

**Results:**

We leverage a dataset of 170 behavioral, physiological, and morphological phenotypes measured in 1812 genetically heterogeneous laboratory mice to study IGE arising between same-sex, adult, unrelated mice housed in the same cage. We develop and apply methods for igeGWAS in this context and identify 24 significant IGE loci for 17 phenotypes (FDR < 10%). We observe no overlap between IGE loci and DGE loci for the same phenotype, which is consistent with the moderate genetic correlations between DGE and IGE for the same phenotype estimated using polygenic models. Finally, we fine-map seven significant IGE loci to individual genes and find supportive evidence in an experiment with a knockout model that *Epha4* gives rise to IGE on stress-coping strategy and wound healing.

**Conclusions:**

Our results demonstrate the potential for igeGWAS to identify IGE genes and shed light into the mechanisms of peer influence.

**Supplementary Information:**

The online version contains supplementary material available at 10.1186/s13059-021-02415-x.

## Background

**Fig. 1:**
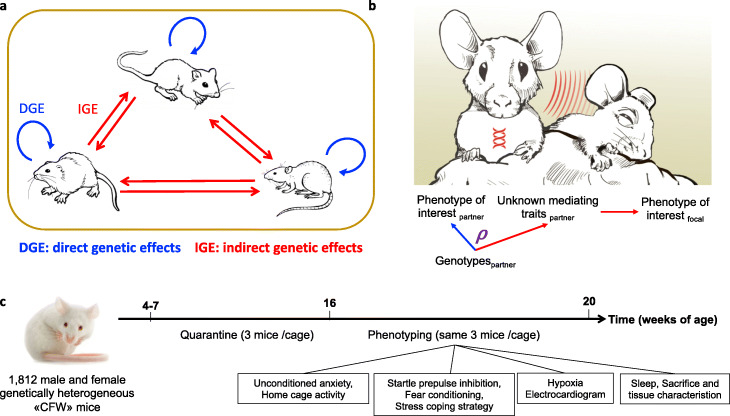
Definition of direct and indirect genetic effects and experimental design. **a** Direct genetic effects (DGE, blue) on an individual’s phenotype arise from the individual’s own genotypes; indirect genetic effects (IGE, red) arise from genotypes of interacting partners (cage mates). This panel illustrates a situation where all individuals are genetically heterogeneous and both DGE and IGE arise from each individual’s genotypes. **b** IGE on a phenotype of interest arise when two individuals interact and (unknown) heritable traits of one individual, the social partner, influence the phenotype of interest measured in the other individual, the focal individual. For a given phenotype of interest, the correlation ρ between DGE and IGE is equivalent to the correlation between DGE on the phenotype of interest and DGE on the traits mediating IGE on the phenotype of interest. Importantly, this correlation can be estimated even when the traits mediating IGE are not known or not measured. **c** Experimental design. A list of the 170 phenotypes collected on each mouse is presented in Additional file 3: Table S2

The phenotype of an individual can be affected not only by the individual’s own genotypes (direct genetic effects, DGE) but also by environmental factors, including the genotypes of other, interacting individuals (indirect genetic effects, IGE) [1–3] (Fig. 1a). IGE arise when the phenotype of a focal individual is influenced by heritable traits of interacting partners (Fig. 1b), which can include behavioral and non-behavioral traits of partners as well as modifications of the non-social environment by partners [4]. IGE have been detected in many laboratory systems [5–14], livestock [15–17], crops [18], wild animals [19–21], and humans [22–27], demonstrating that they are an important component of the genotype to phenotype path and an aspect of the environment that can be studied using genetic approaches.

Most prior studies of IGE have used polygenic modeling approaches to study aggregate genetic effects (i.e., sum of IGE across the genome), either focusing on IGE mediated by *specific traits of partners* using trait-based models [2, 28] or polygenic risk scores [22, 25], or detecting IGE mediated by *unknown heritable traits of partners* using variance component models [9, 15, 29, 30]. More recently, the genome-wide association study of IGE (igeGWAS) has been proposed as a strategy to identify individual genetic loci underlying IGE [5, 7, 8, 11, 31–35].

However, igeGWAS has only been applied in limited settings: in particular, it has not been used to study non-familial IGE from peers affecting biomedical phenotypes, despite growing evidence from polygenic models in laboratory mice [9] and in humans [25, 26] that such effects are important. Moreover, the relationship between DGE and IGE affecting the same phenotype has not been fully addressed, such that the scope for igeGWAS to identify loci not detected by dgeGWAS is unknown. Finally, the results of igeGWAS have not yet been translated into experimentally validated genes causing IGE.

To address these issues, we leveraged a published dataset of 170 behavioral, physiological, and morphological phenotypes measured in 1812 male and female, genetically heterogeneous mice (Fig. 1c), which we supplemented with previously unreported cage information (Additional file 1: Table S1). For each phenotype, we investigated the relationship between DGE and IGE, using both polygenic analyses and GWAS. For 17 phenotypes, we fine-mapped IGE loci to identify putative causal genes underlying IGE. Finally, we tested one such gene using a knockout model.

## Results

We used the genome-wide genotypes (both LD-pruned and unpruned genotypes derived from low-coverage (0.15x) Illumina sequencing, see the “Methods” section) and 200 phenotypes of 1934 commercially available, outbred Crl:CFW(SW)-US_P08(36) (hereafter CFW) mice reported in Nicod et al. [36] and Davies et al. [37]. In addition, we used previously unreported cage information provided by the authors of the original study upon request (Additional file 1: Table S1). Mice were housed in same-sex groups of three and interacted for at least 9 weeks before phenotyping. We excluded any animal whose cage mates changed over the course of the experiment, as well as suspected siblings to rule out confounding from parental and litter effects. These steps resulted in a final sample size of 1812 mice (927 females, 885 males) for analysis. We normalized each phenotype and excluded 30 phenotypes that could not be satisfactorily normalized (see the “Methods” section), yielding a total of 170 phenotypes measured in between 844 and 1729 mice.

### Polygenic analysis of the correlation between DGE and IGE

**Fig. 2:**
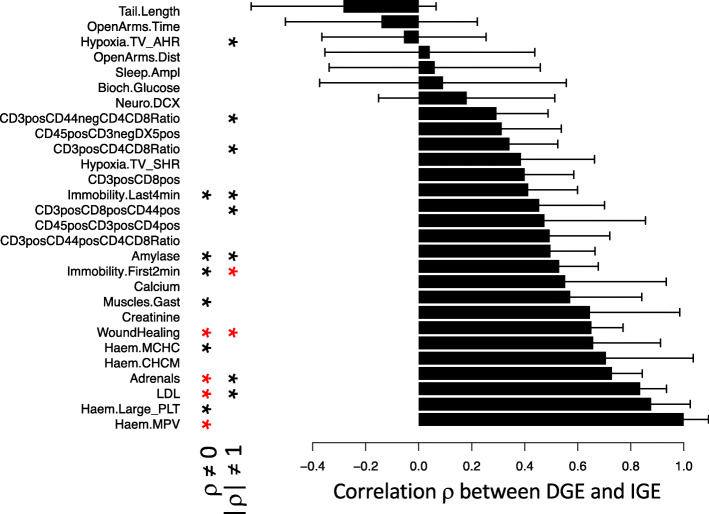
Correlation coefficients ρ between DGE and IGE for the same phenotype, estimated using polygenic models. ρ is shown for 28 phenotypes with marginal DGE and IGE greater than 5%. Error bars denote standard errors. Asterisks on the left show phenotypes for which ρ is significantly different from 0 (black: P < 0.05, red: Bonferroni-corrected P < 0.05). Asterisks on the right show phenotypes for which ρ is significantly different from ±1 (i.e., |ρ| different from 1) (black: P < 0.05, red: Bonferroni-corrected P < 0.05). Numerical values are provided in Additional file 3: Table S2

Initially, we used polygenic models to assess the extent to which loci are shared between DGE and IGE affecting the same phenotype. Briefly, for each trait, we estimated the genetic correlation *ρ* between DGE and IGE. As this correlation is equivalent to the correlation between DGE on the phenotype of interest and DGE on the traits mediating IGE (Fig. 1b), a correlation coefficient of 0 would indicate that the traits mediating IGE are genetically uncorrelated (in the classical sense) to the phenotype of interest, whereas a correlation coefficient of ±1 would indicate that the phenotype of interest itself mediates IGE. For 28 traits with evidence for marginal aggregate DGE and IGE (> 5% of phenotypic variance explained; Additional file 2: Fig. S1), we tested whether *ρ* is equal to 0 and whether it is equal to ±1 (Fig. 2 and Additional file 3: Table S2). We found that *ρ* is different from 0 for 10 out of 28 phenotypes (P < 0.05), indicating that, often, the traits mediating IGE on a phenotype of interest are genetically correlated (in the classical sense) with the phenotype of interest. Evidence that *ρ* is different from zero was strongest for mean weight of the adrenal glands, which correlates with stress [38], mean platelet volume, LDL cholesterol levels, and rate of healing from an ear punch. Second, *ρ* is different from ±1 for 10 phenotypes (P < 0.05), with the strongest evidence for a measure of stress-coping strategy (immobility in the forced swim test) and rate of healing from an ear punch. These results indicate that IGE on a phenotype of interest are often mediated by traits of partners different from the phenotype of interest. To uncover those traits, we turned to igeGWAS.

### igeGWAS and dgeGWAS of the same 170 phenotypes

Next, to compare DGE and IGE at the level of individual loci, we considered the LD-pruned set of variants and performed igeGWAS and dgeGWAS in an analogous manner for each of the 170 phenotypes. For igeGWAS, we estimated the “social genotype” of a mouse at a variant as the sum of the reference allele dosages across its two cage mates at the variant [31, 39] and tested for association between this social genotype and the phenotype of interest. To avoid spurious associations, we accounted for background IGE, background DGE, and shared environmental (cage) effects using random effect components in a linear mixed model (see the “Methods” section). Additionally, we included a fixed effect covariate for DGE arising from the tested variant in igeGWAS. This approach accounts for correlations between direct and social genotypes arising when each individual serves as both focal individual and social partner in the analysis, a strategy that maximizes sample size when all the individuals are genotyped and phenotyped [31, 39]. Accounting for such correlations was required to obtain appropriately calibrated P values in our cohort (Additional file 2: Fig. S2), and theoretical considerations show that it is required even when considering strictly unrelated samples (Additional file 2: Supplementary Note). Finally, we adapted previous strategies [36, 40, 41] based on genome-wide permutations to control the per-phenotype FDR (see the “Methods” section), thereby accounting for the specific patterns of linkage disequilibrium present in the sample.

igeGWAS identified a total of 24 significant loci across 17 of the 170 tested phenotypes (FDR < 10%), including measures relevant to behavior, adult neurogenesis, blood biochemistry, red and white blood cells, apparent bone mineral content, electrocardiography, and ventilatory responses to acute hypoxia (Additional file 4: Table S3). The 17 phenotypes with one or more significant IGE loci tended to have a higher aggregate contribution of IGE than phenotypes without significant IGE loci (averages of 3.8% and 2.8%, respectively), a trend that was not significant (one-sided t-test P = 0.14).

To enable a direct comparison between igeGWAS and dgeGWAS, we performed dgeGWAS for each phenotype using the same approach as taken for igeGWAS, including random effects for DGE and IGE polygenic effects and cage effects and including a fixed effect covariate for IGE arising from the tested variant. This identified 120 significant DGE loci for 63 phenotypes (FDR < 10%; Additional file 5: Table S4). Consistent with the difference in the number of discoveries, we observed that significant IGE loci had, on average, lower effect sizes (proportion of phenotypic variance explained) than DGE loci (Additional file 2: Fig. S3). In light of the observed effect sizes and due to the winner’s curse (or Beavis effect [42, 43]), we expect a larger proportion of significant IGE loci to be false associations compared to significant DGE loci.

**Fig. 3:**
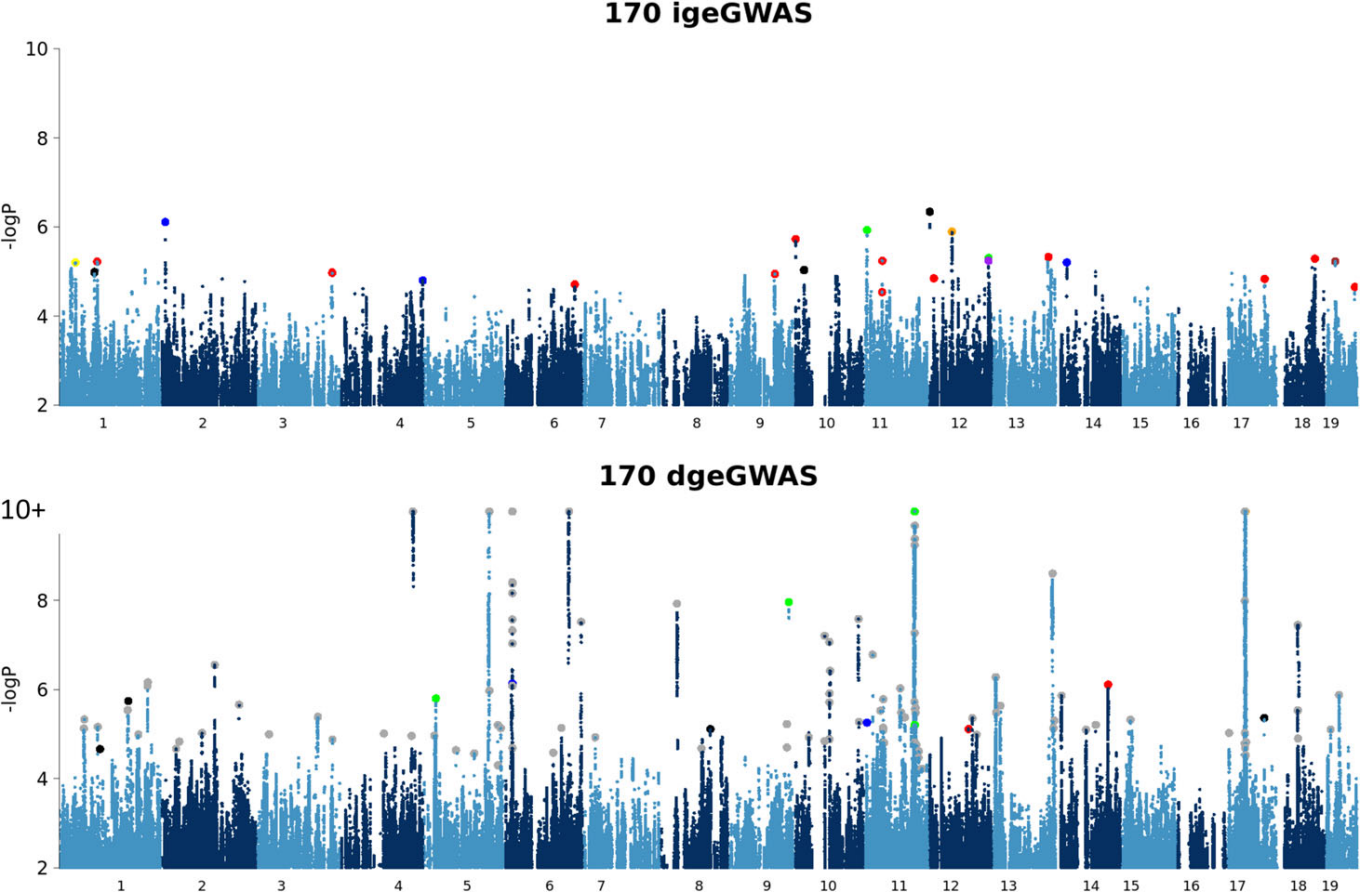
Superimposed Manhattan plots corresponding to igeGWAS (top panel) and dgeGWAS (bottom panel) of the same 170 phenotypes. DGE associations with a negative log P value greater than 10 were truncated at this threshold (as indicated by 10+); also, data points with negative log P values smaller than 2 are not shown. The larger dots correspond to the most significant variant at each significant IGE or DGE locus (FDR < 10%). In the IGE panel (top), each color corresponds to a class of phenotypes: behavioral (red, includes seven behavioral phenotypes with a significant IGE locus), adult neurogenesis (black, two phenotypes with a significant IGE locus), immune (orange, one phenotype with a significant IGE locus), hematological (yellow, one phenotype with a significant IGE locus), blood biochemistry (blue, two phenotypes with a significant IGE locus), bone phenotypes (green, two phenotypes with a significant IGE locus), heart function (brown, one phenotype with a significant IGE locus), and lung function (purple, one phenotype with a significant IGE locus). In the DGE panel (bottom), the same coloring scheme is used as in the IGE panel except for gray dots, which are for phenotypes that do not have any significant IGE locus

There was no overlap between significant DGE and IGE loci for the same phenotype, or even for related phenotypes (Fig. 3, Additional file 4: Table S3 and Additional file 5: Table S4). This observation was expected based on the moderate values observed for the correlation *ρ* between DGE and IGE and the limited power of dgeGWAS and igeGWAS. However, we identified further reason why dgeGWAS and igeGWAS might identify different loci: using simulations to identify key parameters determining the power of igeGWAS, we found that both the number of cage mates and the mode of aggregation across cage mates (i.e., whether the IGE received by a focal mouse correspond to the sum or the average of the IGE emitted by its cage mates) are important, in addition to the parameters also determining the power of dgeGWAS, namely minor allele frequency (MAF) and allelic effect (Additional file 2: Fig. S4). Thus, for a given MAF, allelic effect and a number of cage mates equal to two as is the case in this study, dgeGWAS is expected to have greater power than igeGWAS if IGE get averaged across the two cage mates, but igeGWAS is expected to have greater power than dgeGWAS if IGE sum up across the two cage mates. Importantly, the mode of aggregation across cage mates (sum or average) can be different for different phenotypes. As sample sizes increase for dgeGWAS and igeGWAS, the moderate genetic correlation *ρ* between DGE and IGE and the differences in power between dgeGWAS and igeGWAS dictated by the number of cage mates and the mode of aggregation across cage mates will continue to drive the identification of different loci by dgeGWAS and igeGWAS.

### Identification of putative causal genes for experimental evaluation

Linkage disequilibrium decays faster in the CFW population than in many other mouse populations used for mapping, which facilitates identification of putative causal genes at associated loci [36, 44, 45]. To identify such genes, we fine-mapped the 24 significant IGE loci using the full set of variants (rather than the pruned set used for igeGWAS) in the 1.5-Mb window surrounding the most significant variant at the locus, which corresponds, in this sample, to the average 95% confidence interval for the association [36]. We then identified, for each significant IGE locus, all the genes that either overlap the associated plateau or are located in direct proximity (see the “Methods” section, genes listed in Additional file 4: Table S3 and local association plots available from figshare [46]). At seven loci, there was a single putative causal gene: *Abca12* at a locus for adult neurogenesis; *Epha4* (stress-coping strategy); *Pkn2*, *Slit3*, and *Pgk1-rs7* (at three different loci for sleep); *H60c* (home cage activity); and *Adcy1* (osteopetrosis).

**Fig. 4:**
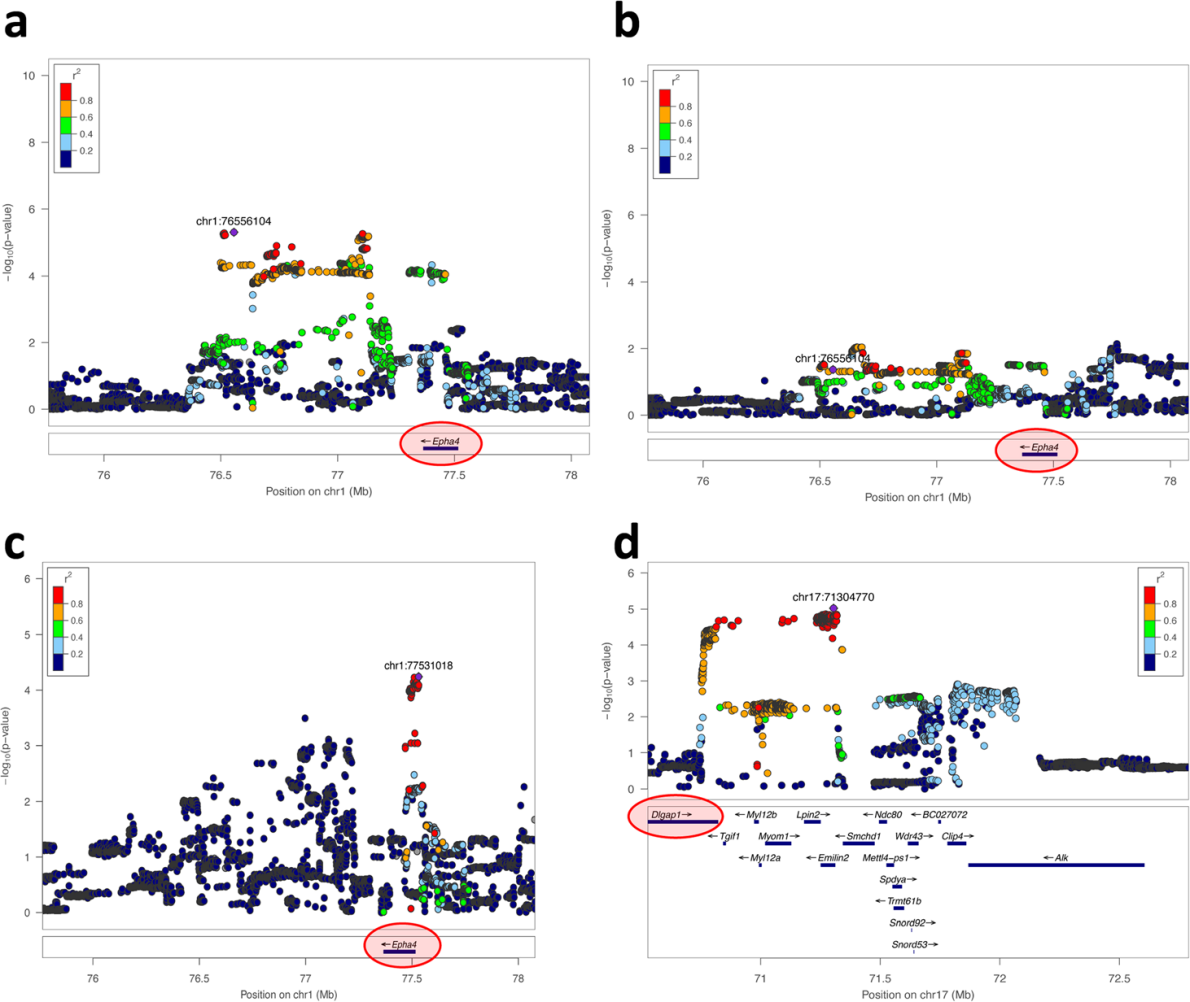
Locus zoom plots for the four associations in CFW outbred mice that were subsequently tested in an experiment with *Epha4* and *Dlgap1* knockout models. **a** Significant IGE locus on chromosome 1 for immobility during the first 2 min of the forced swim test (FST), a measure of stress-coping strategy. *Epha4* was identified as the only putative causal gene at this locus (see the “Methods” section). **b** Same locus and phenotype but DGE, rather than IGE, are shown. The plot shows little evidence of DGE on FST immobility at the *Epha4* locus. **c** Suggestive IGE association at the *Epha4* locus with rate of healing from an ear punch, a phenotype of particular interest (-logP = 4.1, FDR > 10%). **d** Second significant (FDR < 10%) IGE locus for immobility in the FST (this time measured during the last 4 min of the test). *Dlgap1* is one of eight putative causal genes at this locus. It was singled out because of its functional similarity and co-expression with *Epha4* (see main text)

One example of a putative causal IGE gene identified via this strategy is *Epha4*, which was identified at an IGE locus on chromosome 1 for immobility during the first 2 min of the forced swim test (FST), a measure of stress-coping strategy [47] (Fig. 4a and Additional file 2: Fig. S5). We focused on *Epha4* initially because it was the only putative causal gene at a significant locus, the locus was in the top half of the list in terms of significance, and a knockout mouse model was readily available from a neighboring institute. In addition, *Epha4* is in a gene desert and the only protein-coding gene in a chromatin topological domain [48, 49], meaning the causal variant(s) at the *Epha4* locus likely affect the expression of *Epha4* and not that of a neighboring gene.

*Epha4* encodes a synaptic protein that plays an important role in synaptic plasticity in the hippocampus [50, 51] and DGE of *Epha4* on FST immobility have been reported [52, 53]. Therefore, we evaluated the possibility that *Epha4* directly influences stress-coping strategy and that the stress-coping strategy of a mouse in the weeks prior to or during the FST gets copied by the other mice in the cage (behavioral contagion), thereby giving rise to IGE on stress-coping strategy. To investigate this hypothesis, we tested whether *Epha4* has direct effects on FST immobility in CFW mice, using the full set of variants in the same 1.5-Mb window including *Epha4* as for IGE analysis. We found little evidence that *Epha4* directly affects FST immobility in CFW mice (maximum -logP value at the locus. 2.14, Fig. 4b), making it unlikely that behavioral contagion explains the detected IGE in CFW mice.

In addition to the significant IGE association between *Epha4* and FST immobility, we found suggestive evidence for an IGE association between *Epha4* and rate of healing from an ear punch (igeGWAS -logP value = 4.1, FDR > 10%, Fig. 4c). This finding was of particular interest because the *Epha4* locus was among the three most significant IGE loci for wound healing (all three loci with -logP = 4.1, FDR > 10%) and because IGE on wound healing seem to be ubiquitous in laboratory mice: indeed, we have found a significant *aggregate* contribution of IGE to rate of healing from an ear punch in all three mouse populations we have looked at to date (inbred C57BL/6J mice and outbred Heterogeneous Stock mice in Baud et al. [9], and CFW mice in this study). Thus, we were particularly interested in testing whether *Epha4* is involved in IGE on wound healing.

We found two additional significant IGE loci for FST immobility, more precisely for immobility during the last 4 min of the test (Additional file 4: Table S3, Additional file 2: Fig. S6a). At the locus on chromosome 17, we identified eight genes as putatively causal but singled out *Dlgap1* (Fig. 4d) for experimental validation because it encodes a synaptic protein [54], like *Epha4*, and because its expression in the hippocampus, which was measured in a separate cohort of 79 male CFW mice [45], is significantly and highly correlated with that of *Epha4* (Spearman r = 0.868, Bonferroni-corrected P = 2.3·10^-19^, Additional file 2: Fig. S6b). As was the case for *Epha4*, we found no evidence of DGE arising from *Dlgap1* and affecting FST immobility in CFW mice (maximum -logP value at the locus 2.46).

### Evaluating the role of *Epha4* and *Dlgap1* in IGE using knockout models

We tested the hypotheses that *Epha4* can give rise to IGE on FST immobility and rate of healing using a constitutive *Epha4* knockout model on a mixed C57BL/6, C57BL/10 and 129 genetic background. In addition, we tested for IGE from *Dlgap1* on FST immobility using a constitutive *Dlgap1* knockout model on a C57BL/6N background. At weaning, one *Epha4* mouse (heterozygote or wild-type, see the “Methods” section) or one *Dlgap1* mouse (homozygote knockout, heterozygote, or wild-type) was co-housed with one focal FVB/NJ (FVB) mouse of the same sex (male or female). The FVB strain was chosen because it is the *inbred* strain whose genetic background is most similar to that of the *outbred* CFW mice used in igeGWAS, contributing 38% of all alleles in CFW mice [36]. Focal FVB mice were ear punched prior to pairing, then the pairs of mice were left to interact in their cages for two months before they were all tested in the FST and the ears of FVB mice were analyzed to measure the rate of healing (see the “Methods” section).

Although FVB mice are genetically similar to CFW mice, we observed that focal FVB mice showed much less immobility during the first 2 min of the FST than CFW mice (2.0 s on average across all FVB mice vs 12.2 s on average across all CFW mice). Therefore, in our analysis of FVB focal mice, we focused on immobility during the last 4 min of the test, even though this measure showed a less significant association in igeGWAS than immobility during the first 2 min of the test (-logP = 2.8 and 5.2, respectively).

**Fig. 5:**
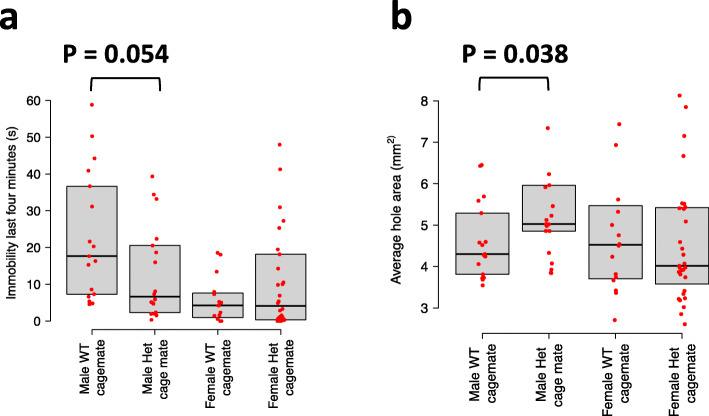
Results of an experiment in which FVB focal mice were co-housed with *Epha4* knockout heterozygote (Het) or wild-type (WT) cage mates in pairs. (**a**) Immobility during the last 4 min of the FST and (**b**) rate of healing from an ear punch were measured in FVB focal mice after 2 months of co-housing

When considering males and females together, we found no effect of the genotype of cage mates on either FST immobility (P = 0.52, ANOVA, N = 81) or wound healing (P = 0.40, ANOVA, N = 85). However, model comparison using the Akaike Information Criterion (AIC) indicated there is an interaction between sex and genotype of the cage mate (i.e., IGE) for both FST immobility and wound healing, as the model including an interaction term between sex and genotype of the cage mate was favored. Therefore, we considered the two sexes separately and observed, in males but not in females, suggestive evidence of IGE on FST immobility (P = 0.054, ANOVA, N = 35) and wound healing (P = 0.038, ANOVA, N = 38) (Fig. 5). The detection of male-specific IGE from *Epha4* on wound healing is consistent with the observation of stronger IGE at the *Epha4* locus in male CFW mice compared to female CFW mice (Additional file 2: Fig. S7a). The detection of male-specific IGE on FST immobility, on the other hand, was not expected from the analysis of CFW mice as similar effects were observed in males and females (Additional file 2: Fig. S7b and S7c). A potential explanation for male-specific IGE on FST immobility in FVB focal mice is that FVB females show lower immobility than FVB males (Fig. 5), hindering our ability to detect genetic effects. Nevertheless, these experimental results support the hypotheses made following igeGWAS that *Epha4* can give rise to IGE on FST immobility and wound healing in laboratory mice.

As was the case in CFW mice, we did not observe a direct effect of *Epha4* on FST immobility whether all mice or males only were considered (P = 0.22 and 0.23, respectively, ANOVA, N = 81 and 35, respectively), indicating behavioral contagion is unlikely to explain these IGE.

Finally, we found no evidence of IGE from *Dlgap1* on FST immobility.

## Discussion

In this study, we leveraged a published dataset of 170 behavioral, physiological, and morphological phenotypes measured in 1812 genetically heterogeneous mice housed in same-sex groups of three to characterize the relationship between DGE and IGE for the same phenotype and identify individual IGE loci. Using polygenic models, we showed that the genetic correlation ρ between DGE and IGE for the same phenotype is often significantly different from ±1, indicating IGE loci are different from DGE loci for the same phenotype. Consistently, we found that none of the 24 significant IGE loci identified for 17 phenotypes using igeGWAS overlapped with significant DGE loci identified for the same phenotypes using dgeGWAS. We fine-mapped seven significant IGE loci to a single putative causal gene and experimentally validated IGE from one of them, *Epha4*, on stress-coping strategy and wound healing using a knockout model.

The analysis of the genetic correlation ρ between DGE and IGE for the same phenotype provides insights into the overlap between DGE and IGE loci for a given phenotype and whether the traits mediating IGE on a phenotype of interest are genetically correlated (in the classical sense) with that phenotype. The correlation ρ was expected to be different from 0 for many phenotypes, based on reports that emotions [55–57], behaviors [25, 58, 59], pathogens, and components of the gut microbiome [60] can “spread” between individuals and contribute to phenotypic variation, both in mice and in humans. In our study, we found that ρ is significantly different from 0 for a variety of phenotypes, which indicates some overlap between DGE loci and IGE loci for the same trait and is consistent with a genetic correlation (in the classical sense) between the phenotype of interest and the traits mediating IGE. However, we also found that ρ is significantly different from ±1 for 10 out of 28 traits, reflecting differences between DGE and IGE loci and demonstrating that IGE on a phenotype of interest often involve traits of cage mates other than the phenotype of interest. This was true even for phenotypes that spread, namely stress and stress-coping strategies. Notably, a similar conclusion has been reached in a study of IGE between spouses in the UK BioBank [26].

Consistent with the estimates of ρ from polygenic models, we found no overlap between the 24 loci identified by igeGWAS for 17 phenotypes and the loci identified by dgeGWAS for the same phenotypes. Our survey of a large number of phenotypes suggests that the loci identified by igeGWAS will, generally, be different from those identified by dgeGWAS, meaning igeGWAS holds great potential to uncover new loci underlying phenotypic variation and that these loci will point to traits of cage mates different from the phenotype studied.

Identifying IGE genes using igeGWAS has been previously attempted [5, 7, 8, 11, 31–35], but there has been limited evidence that this approach can indeed identify genes that are causally involved in IGE. The results of our igeGWAS and fine-mapping analyses identified a single putative causal gene at seven IGE loci: *Abca12* at a locus for adult neurogenesis; *Epha4* at a locus for stress-coping strategy; *Pkn2*, *Slit3*, and Pgk1-rs7 at three different loci for sleep; *H60c* at a locus for home cage activity; and *Adcy1* at a locus for osteopetrosis. Among the loci with a single putative causal gene, the *Epha4* locus stood out because of its relatively high significance and because *Epha4* is the only gene in a well-characterized topological domain [48, 49], meaning the causal variant(s) at the *Epha4* locus likely affect the expression of *Epha4* and not that of a neighboring gene. Hence, we tested *Epha4* in an experiment with a knockout model. Model selection analysis using the AIC indicated an interaction between the *Epha4* allele and the sex of cage mates for both stress-coping strategy and wound healing and, indeed, we found suggestive evidence of IGE from *Epha4* on both phenotypes in male mice (P = 0.054 and 0.038, respectively), but not in females. A limitation of our experiment is that FVB focal mice showed little to no immobility during the first 2 min of the FST, in contrast with the CFW mice used in igeGWAS. Hence, even though the significant igeGWAS locus was for immobility during the first 2 min of the test, we had to focus on immobility during the last 4 min when analyzing the behavior of FVB mice. Similarly, immobility during the last 4 min was lower in FVB female mice than FVB male mice, which may explain why we only observed IGE from *Epha4* in males. Effects of the genetic background of knockout models have been reported in studies of DGE [61]; our results show that in studies of IGE the genetic background of the focal individuals matters too. In the future, we will consider a broader range of genetic backgrounds for focal mice.

The seven genes listed above as single putative causal genes at IGE loci are valuable starting points to gain insights into the mechanisms of IGE. In some cases, it is possible to make testable hypotheses as to the mechanisms mediating IGE from knowledge of the expression profile and function of the gene. For example, *Epha4* plays an important role in the development of the central nervous system and in synaptic plasticity [50, 51], and it has been reported to have DGE on stress-coping strategy [52, 53]. Hence, we hypothesized that IGE from *Epha4* were mediated by behaviours of cage mates, more specifically by focal mice copying the stress-coping strategy of their cage mates (behavioral contagion). We found no evidence of DGE from *Epha4* in either CFW or FVB mice however, suggesting other behaviours of cage mates—or other traits altogether—are involved. As far as wound healing is concerned, the reported expression of *Epha4* in platelets in humans [62], the presence of platelets in saliva [63], and the role of platelets in wound healing and tissue regeneration [64] suggest wound licking by cage mates may be mediating IGE from *Epha4* on wound healing.

Finally, we identified challenges and solutions to different sources of confounding in igeGWAS. In particular, we demonstrated that correlations between direct and social genotypes arise when study individuals play both roles of focal individuals and social partners and that, counter-intuitively, these correlations arise even when all individuals are strictly unrelated. We showed that accounting for direct effects of the locus tested in the null model for igeGWAS permits avoiding spurious IGE associations. These insights, combined with the light we shed on two key parameters determining the power of igeGWAS, namely the number of cage mates and the mode of aggregation of IGE across cage mates, will inform the design and analysis of future igeGWAS.

## Conclusions

Our results demonstrate the potential for igeGWAS to uncover genetic effects expressed only in the context of social interactions and to serve as a starting point for follow-up analyses and experiments that will improve our understanding of peer effects on health and disease.

## Methods

### Phenotypes and experimental variables

Phenotypes and experimental variables (covariates) for 1934 male and female Crl:CFW(SW)-US_P08 (CFW) mice were retrieved from http://wp.cs.ucl.ac.uk/outbredmice/. Phenotypes were normalized using the boxcox function (MASS package [65]) in R; phenotypes that could not be normalized satisfactorily (transformation parameter lambda outside of −2 to 2 interval) were excluded. Because data for some phenotypes were missing for some mice, the sample size varied. The sample size for each phenotype after all filtering (see below) is indicated in Additional file 3: Table S2. The subset of covariates used for each phenotype, which always included sex, is indicated in Additional file 3: Table S2. For those phenotypes where body weight was included as a covariate, we checked that this did not lead to systematically increased (or decreased) estimates of the aggregate contribution of IGE (collider bias).

### Cage information

Mice were 4 to 7 weeks old when they arrived at the phenotyping facility and were housed in same-sex groups of three mice. They were left undisturbed for 9 to 12 weeks during their time in quarantine and spent another 4 weeks together during phenotyping.

Cage assignments were not included in the publicly available dataset but were provided by the authors upon request and are now provided in Additional file 1: Table S1. Cage assignments were recorded at eleven time points throughout the study and showed that a few mice were taken out of their original cages and singly housed, presumably because they were too aggressive. We only included in our analyses mice that had the same two cage mates throughout the experiment. We further excluded a subset of mice based on their genotype-based genetic similarity, as described below.

Finally, all mice were singly housed during the sleep test and until sacrifice a few days later. Hence, we investigated “persistent” IGE on sleep and tissue phenotypes.

### Genome-wide genotypes

From http://wp.cs.ucl.ac.uk/outbredmice/, we retrieved both allele dosages for 7 million variants and allele dosages for a subset of 353,697 high quality, LD-pruned variants (as described in Nicod et al. [37]; genotyping based on sparse 0.15x sequencing data). We used LD-pruned variants for all analyses but the identification of putative causal genes at IGE loci (see below), for which we used the full set of variants.

### Genetic relatedness matrix and exclusion of presumed siblings

The genetic relatedness matrix (GRM) was calculated as the cross-product of the LD-pruned dosage matrix after standardizing the dosages for each variant to mean 0 and variance 1. A few pairs of mice were outliers in the distribution of GRM values, which made us suspect that siblings had been included in the sample even though they were not supposed to be (siblings were excluded by design). To mitigate confounding of DGE and IGE analyses by parental and litter effects, we excluded 19 cages (57 mice) from all analyses.

### Variance component model

The same model as described in detail in Baud et al. [9] was used. Briefly, the model used is the following:

1 $$ {y}_f={X}_f\underset{\_}{b}+{a}_{D,f}+{e}_{D,f}+{Z}_f\underset{\_}{a_S}+{Z}_f\underset{\_}{e_S}+{W}_f\underset{\_}{c} $$

*y*_*f*_ is the phenotypic value of the focal mouse *f*, *X*_*f*_ is a row of the matrix *X* of covariate values, and *b* a column vector of corresponding coefficients. ${a}_{D,f}$ is the additive direct genetic effects (DGE) of *f*. *Z*_*f*_ is a row of the matrix *Z* that indicates cage mates (importantly *Z*_*i*, *i*_ = 0) and $\underset{\_}{a_S}$ the column vector of additive indirect (social) genetic effects (IGE). $\underset{\_}{e_D}$ refers to direct environmental effects (DEE) and $\underset{\_}{e_S}$ to indirect (social) environmental effects (IEE). *W*_*f*_ is a row of the matrix *W* that indicates cage assignment and *c* the column vector of cage effects.

The joint distribution of all random effects is defined as:

$$ \left[\begin{array}{c}\underset{\_}{a_D}\\ {}\underset{\_}{a_S}\\ {}\underset{\_}{e_D}\\ {}\underset{\_}{e_S}\\ {}\underset{\_}{c}\end{array}\right]\sim \mathrm{MVN}\ \Big(\ 0,\left[\begin{array}{ccccc}{\sigma}_{A_D}^2\mathrm{A}\ & {\sigma}_{A_{DS}}\mathrm{A}& 0& 0& 0\\ {}{\sigma}_{A_{DS}}{\mathrm{A}}^T& {\sigma}_{A_S}^2\mathrm{A}& 0& 0& 0\\ {}0\ & 0& {\sigma}_{E_D}^2\mathrm{I}& {\sigma}_{E_{DS}}\mathrm{I}& 0\\ {}0& 0& {\sigma}_{E_{DS}}{\mathrm{I}}^T& {\sigma}_{E_S}^2\mathrm{I}& 0\\ {}0\ & 0& 0& 0& {\sigma}_C^2\mathrm{I}\end{array}\right] $$

where A is the GRM matrix and I the identity matrix.

The phenotypic covariance is:

$$ {\displaystyle \begin{array}{c}{C}_{i,j}=\mathit{\operatorname{cov}}\ \left({y}_i,{y}_j\right)={\sigma}_{A_D}^2\ {A}_{i,j}+{\sigma}_{A_{DS}}\ \left\{\ {\left(A{Z}^T\right)}_{i,j}+{\left(Z{A}^T\right)}_{i,j}\right\}\kern0.5em +\kern0.5em {\sigma}_{A_S}^2\ {\left( ZA{Z}^T\right)}_{i,j}\ \\ {}+{\sigma}_{E_D}^2\ {I}_{i,j}\kern0.5em +{\sigma}_{E_{DS}}\ \left\{\ {\left(I{Z}^T\right)}_{i,j}\kern0.5em +{\left(Z{I}^T\right)}_{i,j}\right\}\kern0.5em +\kern0.5em {\sigma}_{E_S}^2{\left( ZI{Z}^T\right)}_{i,j}\\ {}+{\sigma}_C^2\ {\left( WI{W}^T\right)}_{i,j}\end{array}} $$

When all cages have the same number of mice, as is the case in this study, the non-genetic random effects are not identifiable [15, 66]. An equivalent model can, in that case, be defined as [66]:

$$ {\displaystyle \begin{array}{cc}\mathit{\operatorname{cov}}\ \left({e}_i,{e}_j\right)={\sigma}_E^2\kern0.5em ={\sigma}_{E_D}^2+2{\sigma}_{E_S}^2+{\sigma}_C^2& \mathrm{if}\ i=j\\ {}\mathit{\operatorname{cov}}\ \left({e}_i,{e}_j\right)={\rho}_E{\sigma}_E^2=2\ {\sigma}_{E_{DS}}+{\sigma}_{E_S}^2+{\sigma}_C^2& \mathrm{if}\ i\ne j\ \mathrm{a}\mathrm{nd}\ i\ \mathrm{a}\mathrm{nd}\ j\ \mathrm{share}\ \mathrm{a}\ \mathrm{cage}\\ {}\mathit{\operatorname{cov}}\ \left({e}_i,{e}_j\right)=0& \mathrm{if}\ i\ \mathrm{a}\mathrm{nd}\ j\ \mathrm{a}\mathrm{re}\ \mathrm{in}\ \mathrm{different}\ \mathrm{cages}\end{array}} $$

We checked that both model (1) and this alternative model yielded the same genetic estimates and maximum likelihoods. The alternative model was fitted using the SimplifNonIdableEnvs option.

### Aggregate contributions of DGE and IGE

The aggregate contributions of DGE and IGE were calculated, respectively, as

$$ sampleVar\left({\sigma}_{A_D}^2\ A\right) $$/*sampleVar*(*C*) and $$ sampleVar\left({\sigma}_{A_S}^2\left( ZA{Z}^T\right)\right)/ sampleVar(C) $$,

where *sampleVar* is the sample variance of the corresponding covariance matrix: suppose that we have a vector $\underset{\_}{x}$ of random variables with covariance matrix *M*, the sample variance of *M* is calculated as

$$ sampleVar(M)=\frac{Tr(PMP)}{n-1} $$

*Tr* denotes the trace, *n* is the sample size, and $P=I-\frac{11^{\prime }}{n}$.

The significance of the IGE variance component was assessed using a two-degree of freedom log likelihood ratio (LLR) test (for the variance component and the covariance with DGE). Note that this testing procedure is conservative. The Q value for the aggregate contribution of IGE was calculated for each phenotype using the R package qvalue [67]. Significant IGE contributions were reported at FDR < 10% (corresponding to Q value < 0.1).

### Correlation between DGE and IGE

The correlation *ρ* between $\underset{\_}{a_D}$ and $\underset{\_}{a_S}$ was calculated as:

$$ \rho =\frac{\sigma_{A_{DS}}}{\sigma_{A_D}\times {\sigma}_{A_S}} $$

We tested whether ρ was significantly different from 0 and whether |ρ| was significantly different from 1 using a one-degree of freedom LLR test, which is conservative for the latter test.

### Simulations for Additional file 2: Fig. S1

Phenotypes were simulated based on the genotypes and cage relationships of the full set of 1812 mice. Phenotypes were drawn from model (1) with the following parameters: IGE explaining between 0 and 35.7% of phenotypic variance, DGE explaining 15% of phenotypic variance, ${\rho}_{A_{DS}}$= 0.47, DEE explaining 22% of phenotypic variance, IEE explaining 16% of phenotypic variance, ${\rho}_{E_{DS}}$ = −0.97, and cage effects explaining 26% of phenotypic variance. These variances correspond to the median value of estimates across traits with aggregate IGE and DGE > 5%. After building the phenotypic covariance matrix, the sample variance of the simulations was calculated and used to calculate “realised” simulation parameters from the “target” parameters above. The realised parameters were used for comparison with the parameters estimated from the simulations.

### Models used for igeGWAS and dgeGWAS

To test IGE of a particular variant in igeGWAS, we compared the following two models:

2 $$ {y}_f={X}_f\underset{\_}{b}+{a}_{D,f}+{e}_{D,f}+{Z}_f\underset{\_}{a_S}+{Z}_f\underset{\_}{e_S}+{W}_f\underset{\_}{c}+{G}_f{b}_D $$

3 $$ {y}_f={X}_f\underset{\_}{b}+{a}_{D,f}+{e}_{D,f}+{Z}_f\underset{\_}{a_S}+{Z}_f\underset{\_}{e_S}+{W}_f\underset{\_}{c}+{G}_f{b}_D+{Z}_fG{b}_S $$

Here, *G* is the vector of direct genotypes at the tested variant; hence, *G*_*f*_ is the genotype of the individual that is phenotyped (*f*) and *Z*_*f*_*G* is the sum of the genotypes of the two cage mates of *f*, the “social genotype” of *f* [31, 39]. *b*_*D*_ the coefficient for local DGE and *b*_*S*_ the coefficient for local IGE. Note that *Z*_*f*_ could be defined as the average of the genotypes of the two cage mates of *f*, in which case $\underset{\_}{b_S}$ would be doubled but the igeGWAS P values would remain unchanged. In igeGWAS, we refer to the inclusion of *G*_*f*_*b*_*D*_ in models (2) and (3) as “conditioning.”

The models were fitted with the covariance of the model estimated only once per phenotype, in the model with no local genetic effect (model (1)).

The significance of local IGE was calculated by comparing models (2) and (3) with a 1-degree of freedom LLR test.

dgeGWAS was carried out by comparing model (3) above to the null model (4) below:

4 $$ {y}_f={X}_f\underset{\_}{b}+{a}_{D,f}+{e}_{D,f}+{Z}_f\underset{\_}{a_S}+{Z}_f\underset{\_}{e_S}+{W}_f\underset{\_}{c}+{Z}_fG{b}_S $$

In dgeGWAS, we refer to the inclusion of *Z*_*f*_*Gb*_*S*_ in model (4) and (3) as “conditioning.”

### Identification of significant associations

We used a genome-wide permutation strategy to control the FDR for each phenotype, as done by Nicod et al. [36]. This strategy takes into account the specific patterns of linkage disequilibrium present in the sample and identifies significant associations *for each phenotype independently of the results for the other phenotypes in the dataset*. More precisely, for each phenotype and for each type of genetic effect (direct and indirect), we performed 100 “permuted GWAS” by permuting the rows of the matrix of social (respectively direct) genotypes and testing each variant at a time using the permuted genotypes together with the un-permuted phenotypes, un-permuted covariates, un-permuted GRM, and un-permuted matrix of direct (respectively social) genotypes (for conditioning) [40, 41]. For a given P value x, the per-phenotype FDR can be calculated as:

$$ FDR(x)=\frac{\# loci\ with\ P<x\  in\ permuted\ data\ }{100\times \# loci\ with\ P<x\  in\ unpermuted\ data} $$

We reported those loci with FDR < 10%.

### Definition of putative causal genes at associated loci

At each significantly associated locus, we defined a 1.5-Mb window centered on the lead variant corresponding, in this sample, to the 95% confidence interval for the association [36]. We identified all the variants that segregate in this window based on the full set of 7M variants and reran igeGWAS and dgeGWAS locally using all the variants at the locus. We defined “putative causal genes” as those genes that either overlapped the associated plateau or were located in direct proximity, and whose MGI symbol does not start by “Gm,” “Rik,” “Mir,” “Fam,” or “Tmem” in order to focus on genes with known function and generate more tractable hypotheses on the mechanisms of IGE.

We identified putative causal genes using locusZoom plots [68]. To create them, we used the standalone version of locusZoom (https://genome.sph.umich.edu/wiki/LocusZoom_Standalone). The plots for all 24 significant IGE loci reported in Additional file 4: Table S3 are available from *figshare* [46].

### Gene expression in the hippocampus of an independent sample of CFW mice

Gene expression in the hippocampus of an independent sample of 79 male CFW mice, initially published in Parker et al. [45], was available from GeneNetwork (http://genenetwork.org/) ([69, 70], (https://pubmed.ncbi.nlm.nih.gov/33472826/)). The data are accessible by selecting Mouse as *Species*, CFW Outbred GWAS as *Group*, Hippocampus mRNA as *Type*, and UCSD CFW Hippocampus (Jan17) RNA-Seq Log2 Z-score as *Dataset*. To retrieve the genes whose expression is most highly correlated with that of *Epha4*, we entered “Epha4” in the *Get Any* field. Following the selection of the Epha4 record (click on ENSMUSG00000026235), we used *Calculate Correlations* with Sample r as *Method*, UCSD CFW Hippocampus (Jan17) RNA-Seq Log2 Z-score as *Database*, and Spearman rank as correlation *Type*. Additional file 2: Fig. S6b is obtained by clicking on the value of the correlation between Epha4 and Dlgap1 expression levels (column *Sample rho*).

### Variance explained by a significant association

The variance explained by a significant IGE association was estimated in an extension of model (1) with additional fixed effects for both DGE and IGE of lead SNPs at all significant IGE loci (the lead SNP being the SNP with the most significant P value at the locus in the igeGWAS). After fitting the model, the variance was calculated as:

$$ \frac{\mathit{\operatorname{var}}\left( ZG\hat{b_S}\right)}{\sum \mathit{\operatorname{var}}\left({X}_c\hat{b_c}\right)+\sum \mathit{\operatorname{var}}\left(G\hat{b_D}\right)+\sum \mathit{\operatorname{var}}\left( ZG\hat{b_S}\right)+ sampleVar(C)} $$

where *sampleVar*(*C*) is the sample variance of the covariance matrix in this model.

The variance explained by a significant DGE association was estimated in a similar model but considering all significant DGE associations and calculated as:

$$ \frac{\mathit{\operatorname{var}}\left( ZG\hat{b_D}\right)}{\sum \mathit{\operatorname{var}}\left({X}_c\hat{b_c}\right)+\sum \mathit{\operatorname{var}}\left(G\hat{b_D}\right)+\sum \mathit{\operatorname{var}}\left( ZG\hat{b_S}\right)+ sampleVar(C)} $$

### Simulations for Additional file 2: Fig. S2b and S2c

Phenotypes were simulated based on the genotypes and cage relationships of the 1812 mice. Null phenotypes (no local IGE) were simulated from model (2) as the sum of random effects and local DGE. The following parameters were used for the random effects: ${\sigma}_{A_D}^2=$20 and ${\sigma}_{A_S}^2$ = 20 (which correspond to high polygenic effects in the real data), ${\rho}_{A_{DS}}$= 0.5, ${\sigma}_{E_D}^2$= 30, ${\sigma}_{E_S}^2$= 30, ${\rho}_{E_{DS}}$ = −0.97, and ${\sigma}_C^2$ = 25 (which are close to the median of the corresponding estimates from the real data). Local DGE were simulated at random variants in the genome to account for 20% of the phenotypic variance.

### Simulations for Additional file 2: Fig. S4

Phenotypes were simulated based on the real genotypes but random cages. Phenotypes were simulated as the sum of random and fixed effects using the following models:

$$ {y}_f={X}_f\underline{b}+{a}_{D,f}+{e}_{D,f}+{Z}_f{a}_{\underline{S}}+{Z}_f{e}_{\underline{S}}+{W}_f\underline{c}+{G}_f{b}_D\kern0.5em \mathrm{for}\kern0.5em \mathrm{local}\kern0.5em \mathrm{DGE} $$

$$ {y}_f={X}_f\underline{b}+{a}_{D,f}+{e}_{D,f}+{Z}_f{a}_{\underline{S}}+{Z}_f{e}_{\underline{S}}+{W}_f\underline{c}+{Z}_f{Gb}_S\kern0.5em \mathrm{for}\kern0.5em \mathrm{local}\kern0.5em \mathrm{IGE} $$

The following parameter values were used for the random effects: ${\sigma}_{A_D}^2=$17, ${\sigma}_{A_S}^2$ = 17, ${\rho}_{A_{DS}}$= 0.65, ${\sigma}_{E_D}^2$= 19, ${\sigma}_{E_S}^2$= 15, ${\rho}_{E_{DS}}$ = −0.8, and ${\sigma}_C^2$ = 25. These values correspond to the median estimates for phenotypes with aggregate IGE and DGE > 0.1.

Local DGE and IGE were simulated at variants with low MAF (MAF < 0.05), medium MAF (0.225 < MAF < 0.275), or high MAF (MAF > 0.45). Local IGE were simulated using two alternative generative models: an “additive” model by using *Z* as in model (3) (i.e., filled with 0s and 1s) or an “average” model by using ${Z}^{\prime }=\frac{Z}{N}$, where *N* = 2. In all cases (DGE, additive IGE, and average IGE), we simulated an allelic effect of 0.2, which is similar to the average allelic effect estimated in the igeGWAS. Power was calculated at a genome-wide significance threshold of negative log P 5, which is similar to the significance of associations detected at FDR < 10%.

### Experiment with *Epha4* and *Dlgap1* knockout mice

#### Experimental design

All animal procedures were approved by the Institutional Animal Care and Use Committee of the University of California San Diego (UCSD) and were conducted in accordance with the NIH Guide for the Care and Use of Laboratory Animals. FVB/NJ (hereafter FVB) breeder mice were originally purchased from the Jackson Laboratory (Bar Harbor, MA, USA), then bred on site. *Epha4* knockout mice (allele name: Epha4tm1Byd) on a mixed C57BL/6, C57BL/10 and 129 genetic background, originally created by Dottori et al. [71], were generously donated by Prof. Elena Pasquale (Sanford Burnham Prebys, San Diego, CA, USA) then bred at UCSD. The mouse line C57BL/6 N-Dlgap1<em1(IMPC)Tcp> was made as part of the KOMP2-Phase2 project at The Centre for Phenogenomics, Toronta, Canada. It was obtained from the Canadian Mouse Mutant Repository and bred at UCSD. Breeding from heterozygous parents produced, for *Dlgap1*, wild-type (WT), heterozygote (Het), and homozygote knockout (KO) offspring. For *Epha4*, homozygote knockout offspring usually died before weaning, leaving Het and WT offspring only. Within 3 days of weaning, we paired one focal FVB mouse with either a *Dlgap1* (WT, Het, or KO) or an *Epha4* (WT or Het) cage mate of the same sex (male or female). Immediately prior to pairing, the FVB mice were ear punched on each ear using 2-mm ear punch scissors. Pairs of mice were then left to interact for 2 months before all mice were phenotyped in the forced swim test (FST) and sacrificed and the ears of FVB mice were collected. The sample size was 52 *Epha4* Het mice and 33 *Epha4* WT mice for wound healing; for FST, there were only 48 *Epha4* Het mice as one mouse died during the FST, two mice had to be separated from their cage mate due to fighting in the days before the FST (but their ears were still collected as this did not significantly change the healing time), and during the FST of the fourth mouse, the battery of the camera ran out. A small subset of mice were video recorded in a new enclosure for 24 h a few days before the FST but the data from this pilot project are not reported here. Throughout the experiment, all mice were housed on a 12-h:12-h light-dark cycle, with lights on at 06:00, and all behavioral testing occurred during the light phase of the light-dark cycle.

#### Forced swim test

Following the same protocol as in the CFW study [36], mice were tested in the forced swim test: they were placed for 6 min in 6’’ wide × 12’’ tall glass buckets filled with water at 24–26 °C. Mice were video recorded from the side and their immobility during the first 2 and last 4 min of the test was scored by an observer blind to the genotypes of the black (*Epha4* and *Dlgap1*) mice. The analysis of IGE focused on immobility of FVB mice in the last 4 min of the test as FVB mice are rarely immobile during the first 2 min of the test.

#### Healing from an ear punch

Both ears of FVB mice were punched with a 2-mm-diameter ear punch scissor just before the mice were paired with an *Epha4* or a *Dlgap1* cage mate at weaning. Following the same protocol as in the CFW study [36], the ears were collected 2 months later after sacrifice and stored in 10% buffered formalin phosphate until analysis. To measure the area of the hole, each ear was mounted on a histology slide and photos were taken from a fixed distance. Images were analyzed with the ImageJ software [72] and the average across the two ears calculated.

#### Genotyping

For genotyping *Epha4* mice, tail or ear biopsies were sent to Transnetyx Inc. for genotyping (Transnetyx Genotyping Services, Cordova, TN). Transnetyx Inc. utilize real-time PCR and duplicate sample processing to ensure the accuracy of each mutation. Additionally, Sanger sequencing and gene expression analysis were performed to further validate the results of the Transnetyx assays.

For genotyping *Dlgap1* mice, we used a multiplex PCR with primers: *CCGTAAGTGAAGTCTCCATCAACAG (Fw1), CGGCTAGGATTTCAGAGTTTGTTC* (Fw2) and *CTTCCTCTCCTACACCATCAACAC (Rev1)*, yielding a 308-bp band in the presence of a WT allele and a 392-bp band in the presence of a knockout allele.

#### Statistical analysis

For both FST immobility and wound healing, five fixed effect models were first compared using the Akaike Information Criterion (AIC): a model with intercept only, a model with the sex of the pair (focal animal and cage mate were always of the same sex), a model with the genotype of the cage mate, a model with both sex and genotype of cage mate, and finally a model with main effects of sex and genotype of cage mate and their interaction.

IGE were then tested in males using an analysis of variance (ANOVA) with one degree of freedom.

## Supplementary Information

**Additional file 1: Table S1**. Cages for all mice across their time at the phenotyping facility.

**Additional file 2: Supplementary note**. Correlation ρ between direct and social genotypes arising from using each individual as both focal individual and social partner. **Fig. S1**. Estimation of the correlation ρ between IGE and DGE in simulations. **Fig. S2**. Correlations between direct and social genotypes of CFW mice, and implications for GWAS. **Fig. S3**. Proportion of phenotypic variance explained by significant DGE and IGE loci. **Fig. S4**. Power to detect DGE and IGE associations in simulations. **Fig. S5**. Quantile-quantile (QQ) plot for the igeGWAS P values for Immobility during the first two minutes of the forced swim test. **Fig. S6**. Information relevant to the role of *Dlgap1* in giving rise to IGE on immobility during the last four minutes of the FST. **Fig. S7**. Phenotypes of the outbred CFW mice used in igeGWAS.

**Additional file 3: Table S2**. Information about each phenotype, aggregate contributions of IGE and DGE, and correlation ⍴ between IGE and DGE.

**Additional file 4: Table S3**. Genome-wide significant IGE associations (per-phenotype FDR < 10%).

**Additional file 5: Table S4**. Genome-wide significant DGE associations (per-phenotype FDR < 10%).

**Additional file 6:.** Review history.

## Data Availability

Genotype and phenotype data from Nicod et al. [36] and Davies et al. [37] are available from http://wp.cs.ucl.ac.uk/outbredmice/. Cage information is provided in Additional file 1: Table S1. CFW hippocampus data is available in GeneNetwork (https://genenetwork.org) as FAIR data and can be analysed reproducibly using the online GWAS web-service. GeneNetwork contains over 20-years of experimental mouse and rat data. The code used for variance decomposition and GWAS in this study is available from https://github.com/Baud-lab under an Apache 2.0 license and from zenodo [73, 74].
