# Supplementary material for: Dissecting indirect genetic effects from peers in laboratory mice
Source: Genome Biol. 2021 Jul 26;22:216. doi: 10.1186/s13059-021-02415-x (PMC8311926; doi:10.1186/s13059-021-02415-x)
Supplement: Supplementary file 6 — Additional file 6:. Review history. [file 13059_2021_2415_MOESM6_ESM.docx]

Review History

**First round of review**

**Reviewer 1**

**Are you able to assess all statistics in the manuscript, including the appropriateness of statistical tests used?**

No, I do not feel adequately qualified to assess the statistics.

**Comments to author:**

The authors have undertaken a very large (number of mice and phenotypes) study to address the potential for social genetic effects (SGE) in co-caged male pairs in relation to direct genetic effects (DGE). This extends prior work and addresses an interesting set of questions. The simulations and methods seem appropriately applied. The writing is overall excellent. The finding of fewer SGE loci of generally smaller effect size seems intuitive and an important result in the field. The highlighted SGE loci are interesting and make some sense in terms of socially affected genetic effects (i.e., sleep, helplessness measure). There are a few larger comments to potentially address and more minor comments.

Major comments

1)	Page 5 - when referring to Baud et al. (5) and discussing results of LDL, wound healing, weight of adrenal glands, hypoxic response - at first I wondered whether the discussion referred to a Supplementary Table 1 in Baud et al. (5) and not the current manuscript? The reason for the confusion is that none of the described phenotypes appear to have significant SGE effects in Supplementary Table 2, and these phenotypes are not really further discussed in the Results and Discussion. There are no DGE effects reported in Table S3 for mean platelet volume, ear punch healing, or weight of adrenal glands. I think more explanation is required for readers why this is the case since these examples are initially highlighted here. Is it that these phenotypes simply had borderline associations that did not reach FDR significance? It then seems a bit incongruent to hold them up as examples of SGE/DGE effects unless I am simply missing something. Overall, I think some clearer discussion (comparison and contrast) from the current and prior B6/D2 study is needed at some point in the manuscript.

2)	Re: Allele freq - it seems there are more rare variants associated in SGE as opposed to DGE. Nearly 50% of the SGE FDR 10% peak variants have MAF <10%, whereas the proportion for DGE with MAF <10% is around 25%. When I plot -logP vs. allele frequency there seems to be an apparent difference. (the Alk_phos DGE locus is an outlier relative to others) There is some bias for common variant effects in DGE (and many of the largest effect size loci being DGE with high allele frequency). Does this indicate something different about the genetic architecture of SGE hits? The authors have discussed some of the differences on P.13-14, and in simulations in Figure 5. Overall, I think the author discussion is relatively good here. However, I would posit another explanation of the empirical data is that you may have higher false positive rates among your SGE loci here (explanations could be lack of true loci, different power, limited cagemate exposure to detect as mentioned, etc.). Given the lower MAF strata and effect size I think this is likely a valid explanation. I think there are parallels in human GWAS mapping studies whereby large effect common variants were found first, and had higher replication rates and validity than rare variant effects. It may be worth reviewing the relevant human GWAS/exome/WGS genetic architecture literature and considering a few statistical citations in discussing. Without attempts to replicate it is more difficult to assess specifically - though replicating SGE/DGE is likely outside the scope of the current study.

Minor comments

1. There is one important biological dynamic that is missing from the current research (and I expect other studies in the SGE field). That is the possible influence of male-female social dynamics, as well as how those dynamics could affect male-male dynamics and SGE. I am not faulting the authors or suggesting they embark on further studies for the current manuscript (obviously designing and dealing with male-female and male-male in the presence of females, influence of female cycles, etc. would be much more complex in design). I think it would just worth briefly mentioning as a potential limitation of the work (and possibly the field in general though I have not reviewed that literature)

2. I wonder if many of the phenotypes explored may be influenced by diet (amount of chow consumed), and if feeding habits may be social (and possibly SGE/DGE) influenced? We know there is strong social influence in human diets from social network studies. Is chow consumption (weight) monitored by cage? I guess this could not be determined on a per mouse basis in the current design? Again, this may just be a potential discussion point or consideration in future study designs if chow could be tracked on a per mouse basis (or some cage/chow sub analyses conducted)

3. The ear wound healing observation, and mean platelet volume observation was interesting to me. It made me wonder if there may be platelet and/or other coagulation link between loci? Given the inverse correlation of MPV and PLT at least in humans (and often shared loci) it made me also wonder if the signals for MPV are not also somewhat shared, if not reaching genome-wide significance for PLT. However, it seems there were no significant SGE effects for either in Table S2 so I guess this cannot be determined at this time.

4. The authors used outbred CFW mice (and mention that LD decays more rapidly in this strain). Can the authors provide a bit more detail on the strain selection rationale and its background/origination - could just be brief comment potentially even in the Methods.

5. Genome-wide genotypes previously published and cited - 7 million variants and LD-pruned set - assumed the origin is WGS but please briefly specify the data origin and platform/type

6. Should there be citations in structured abstract?

7. Figure 2 - would be nice if you could label the phenotypes in small type

8. Color coding of the phenotype categories in Fig 3 does not really add much so I'm not sure it is necessary

9. Supplementary note - please write out iid at first use

10. P. 25 - capitalize "parker"

11. Table captions and/or footnotes/abbreviations would be helpful for the Supplemental Tables. For example it is unclear what things like Weight.Diss, Weight.Hypo, etc. mean - is this weight at dissection? Weight of hypothalamus? hypoxia? What are NB_slices? Many of the phenotypes and abbreviations are intuitive but not all. The Weights are listed at bottom but not intuitive when viewed as covariates at the top of the Table. Is the phenotype "NA" in Row 33 a mistake?

12. Table S2: normalize font size. Please give mouse genome ref build. How were candidate genes at locus selected -- by some distance or linkage bounds? Is this the 1.5 Mb mentioned in the text uniformly applied?

13. Table S3: as in Table S2 similar comments

14. Table S4: label column A as 'Mouse ID'. It is unclear what the abbreviations in Row 4 indicate - please add to sub-caption/footnote. Mice with "NA" - does this indicate that mice were deceased and not carried forward in the study, or otherwise incapacitated/unable to be tested? If so, please indicate (some mice are NA for all cage conditions). I think it is commendable that the authors have provided this level of detail and data availability to enable future researchers to either reconstruct analyses or attempt additional analyses.

**Reviewer 2**

**Are you able to assess all statistics in the manuscript, including the appropriateness of statistical tests used?**

Yes, and I have assessed the statistics in my report.

**Comments to author:**

Baud et al describe the genetic architecture of direct (DGE) and social genetic (SGE) effects for 170 traits measured in outbred mice. They report 24 and 120 genome-wide associated loci for SGE and DGE, respectively. The paper is well written and the general area is of interest but the manuscript has two main issues:

1) there is not attempt to replicate the findings or understand their functional significance. Without replication it is impossible to assess to what degree the reported loci are false positives.

2) the authors, who are worried about having related mice in the same cage, end up using a very ad-hoc approach to avoid removing 435 mice. Whilst I can understand that they want to make a compromise between removing the effect of relatedness and losing statistical power. The truth is that the reader ends up not knowing whether the effect would still persist if the related individuals were removed. In a way, that the genotype of a group of individuals explain variation of their cousins is not that surprising, and keeping only a few cousins could lead to that effect. The authors could perhaps try to see if the effects hold after removing the 435 mice.

Some of the methods are a bit unclear, for instance, how the genotypes of the two mates in a cage are combined into a single component of variance.

On a minor, one of the formula got very weird (page 21).

**Authors Response**

**Point-by-point responses to the reviewers’ comments:**

*We thank you and the reviewers for constructive comments. We have carefully revised the paper (GBIO-D-19-00270) to comprehensively address the comments raised. Specifically we have:*

*·	Included novel experimental data to assess two putative causal genes identified via igeGWAS, namely Epha4 and Dlgap1. Our experimental results confirm that Epha4 gives rise to IGE on both stress-coping strategy and wound healing in laboratory mice. This is the first time findings from igeGWAS have been confirmed by experimental manipulation.*

*·	Repeated all our analyses after excluding all 435 mice showing a higher than expected level of genetic similarity with another mouse in the dataset. These analyses confirmed that the results reported in our manuscript, which were obtained by excluding a subset of 57 of these mice, are unbiased.*

*·	Simplified the manuscript by removing the section describing the aggregate contributiong of IGE to phenotypic variation, in order to focus on novel analyses, namely the analysis of the genetic correlation between DGE and IGE and igeGWAS. We still provide, however, all the data as presented previously (now in Supplementary Table 2).*

*A point by point response to the reviewers’s comments is provided below. We hope our paper is now acceptable.*

*Best wishes,*

*Amelie Baud and Oliver Stegle*

Comments from Reviewer #1:

The authors have undertaken a very large (number of mice and phenotypes) study to address the potential for social genetic effects (SGE) in co-caged male pairs in relation to direct genetic effects (DGE). This extends prior work and addresses an interesting set of questions. The simulations and methods seem appropriately applied. The writing is overall excellent. The finding of fewer SGE loci of generally smaller effect size seems intuitive and an important result in the field. The highlighted SGE loci are interesting and make some sense in terms of socially affected genetic effects (i.e., sleep, helplessness measure). There are a few larger comments to potentially address and more minor comments.

Major comments

1)	Page 5 - when referring to Baud et al. (5) and discussing results of LDL, wound healing, weight of adrenal glands, hypoxic response - at first I wondered whether the discussion referred to a Supplementary Table 1 in Baud et al. (5) and not the current manuscript? The reason for the confusion is that none of the described phenotypes appear to have significant SGE effects in Supplementary Table 2, and these phenotypes are not really further discussed in the Results and Discussion. There are no DGE effects reported in Table S3 for mean platelet volume, ear punch healing, or weight of adrenal glands. I think more explanation is required for readers why this is the case since these examples are initially highlighted here. Is it that these phenotypes simply had borderline associations that did not reach FDR significance? It then seems a bit incongruent to hold them up as examples of SGE/DGE effects unless I am simply missing something. Overall, I think some clearer discussion (comparison and contrast) from the current and prior B6/D2 study is needed at some point in the manuscript.

*The section the Reviewer is referring to used to describe the aggregate contribution of SGE (called IGE in our revised manuscript) to phenotypic variation, in other words the sum of IGE across all variants in the genome. The results were originally reported in Supplementary Table 1.*

*LDL, wound healing, weight of adrenal glands, hypoxic response all have significant aggregate IGE (FDR < 10%). In our revised manuscript, however, we do not discuss the aggregate contribution of IGE to phenotypic variation any more in the main text, as this is not the focus of this study (it was the focus of Baud et al. 2017). We still report the same results as before, but they are now in Supplementary Table 2 of our revised manuscript.*

*2)	Re: Allele freq - it seems there are more rare variants associated in SGE as opposed to DGE. Nearly 50% of the SGE FDR 10% peak variants have MAF <10%, whereas the proportion for DGE with MAF <10% is around 25%. When I plot -logP vs. allele frequency there seems to be an apparent difference. (the Alk_phos DGE locus is an outlier relative to others) There is some bias for common variant effects in DGE (and many of the largest effect size loci being DGE with high allele frequency).*

Does this indicate something different about the genetic architecture of SGE hits? The authors have discussed some of the differences on P.13-14, and in simulations in Figure 5. Overall, I think the author discussion is relatively good here. However, I would posit another explanation of the empirical data is that you may have higher false positive rates among your SGE loci here (explanations could be lack of true loci, different power, limited cagemate exposure to detect as mentioned, etc.). Given the lower MAF strata and effect size I think this is likely a valid explanation. I think there are parallels in human GWAS mapping studies whereby large effect common variants were found first, and had higher replication rates and validity than rare variant effects. It may be worth reviewing the relevant human GWAS/exome/WGS genetic architecture literature and considering a few statistical citations in discussing. Without attempts to replicate it is more difficult to assess specifically - though replicating SGE/DGE is likely outside the scope of the current study.

*We agree with this Reviewer that associations with smaller effect sizes (proportion of phenotypic variance explained) are more likely to be false positives, as a result of the winner’s curse. We have added a statement to this effect in our revised manuscript: “Consistent with the difference in the number of discoveries, we observed that significant IGE loci had, on average, lower effect sizes (proportion of phenotypic variance explained) than DGE loci (Supplementary Figure 3). In light of the observed effect sizes and due to the winner’s curse (or Beavis effect (37, 38)), we expect a larger proportion of significant IGE loci to be false associations, compared to significant DGE loci.”*

*We do not think, however, that the lower average MAF of IGE loci compared to DGE loci is indicative, per se, of a greater rate of false positives among IGE loci. Indeed, whereas the power to detect an association (both for DGE and IGE) depends on MAF and allelic effect of the variant, the power to detect IGE associations also depends on the number of cage mates and the mode of aggregation across cage mates (i.e. whether the IGE received by a focal mouse correspond to the sum or the average of the IGE emitted by its cage mates), as we show using a power analysis discussed in the main text and presented in Supplementary Figure 4. Therefore, the lower MAF of IGE loci compared to DGE loci could be compensated for by amplification (addition) of the IGE signal across the two cage mates.*

*In human genetics, replicating an association in an independent cohort is key to establishing the robustness of a GWAS locus. Unfortunately, and as we detail in our reponse to Reviewer 2 below, no appropriate CFW cohort was available for replicating the IGE loci we identified in this study. What we did, instead, was to conduct a follow up experiment with a knock-out model to evaluate the role of a putative causal gene, Epha4, identified at an IGE locus for stress-coping strategy. The results of this experiment lended support to the hypothesis that Epha4 gives rise to IGE on stress-coping strategy, supporting the validity of the igeGWAS approach we used to identify Epha4 and our ability to identify genuine IGE signals. Our experimental results also mark the first step towards a more detailed experimental dissection of the mechanisms underlying IGE from Epha4.*

Minor comments

1.	There is one important biological dynamic that is missing from the current research (and I expect other studies in the SGE field). That is the possible influence of male-female social dynamics, as well as how those dynamics could affect male-male dynamics and SGE. I am not faulting the authors or suggesting they embark on further studies for the current manuscript (obviously designing and dealing with male-female and male-male in the presence of females, influence of female cycles, etc. would be much more complex in design). I think it would just worth briefly mentioning as a potential limitation of the work (and possibly the field in general though I have not reviewed that literature)

*We have stressed in the Abstract, Results and Methods sections that we have studied “same-sex groups”, to clarify that our results are only relevant to this situation. In the future we plan to study differences in IGE between groups of males and groups of females, and may investigate male-female interactions as well, but this is outside the scope of this study.*

2.	I wonder if many of the phenotypes explored may be influenced by diet (amount of chow consumed), and if feeding habits may be social (and possibly SGE/DGE) influenced? We know there is strong social influence in human diets from social network studies. Is chow consumption (weight) monitored by cage? I guess this could not be determined on a per mouse basis in the current design? Again, this may just be a potential discussion point or consideration in future study designs if chow could be tracked on a per mouse basis (or some cage/chow sub analyses conducted)

*Chow was provided ad-libitum and consumption was not monitored, but body weight was measured at several time points. However, estimates of the aggregate contribution of IGE to these measures were all very low and we did not detect any significant IGE locus either, suggesting there are little to no IGE on body weight in CFW mice.*

3.	The ear wound healing observation, and mean platelet volume observation was interesting to me. It made me wonder if there may be platelet and/or other coagulation link between loci? Given the inverse correlation of MPV and PLT at least in humans (and often shared loci) it made me also wonder if the signals for MPV are not also somewhat shared, if not reaching genome-wide significance for PLT. However, it seems there were no significant SGE effects for either in Table S2 so I guess this cannot be determined at this time.

*We found a large contribution of aggregate IGE to wound healig and MPV, but not for PLT or PCT (Supplementary Table 2 in our revised manuscript). However, the analysis we have employed in this study is univariate (one phenotype at a time), so we cannot speak to the genetic correlation between IGE on wound healing and IGE on MPV.*

*As we did not find any significant IGE loci for wound healing, MPV, PLT or PCT, the analysis of individual loci does not allow us to speak to any link between loci either.*

4.	The authors used outbred CFW mice (and mention that LD decays more rapidly in this strain). Can the authors provide a bit more detail on the strain selection rationale and its background/origination - could just be brief comment potentially even in the Methods.

*We have added to the first paragraph of the Results section of our revised manuscript a more precise description of CFW mice, including their exact stock name, a reference to Yalcin et al PLOS Genetics 2010 describing the origin of this stock, and the fact that these mice are commercially available.*

*We also expanded the first paragraph of the section “Identification of putative causal genes for experimental evaluation” to more explicitly justify our choice of CFW mice: “Linkage disequilibrium decays faster in the CFW population than in many other mouse populations used for mapping, which facilitates identification of putative causal genes at associated loci(32, 33, 40).”*

5.	Genome-wide genotypes previously published and cited - 7 million variants and LD-pruned set - assumed the origin is WGS but please briefly specify the data origin and platform/type

*We have added a sentence to the first paragraph of the Results section to indicate that the genotype data were “derived from low-coverage (0.15×), Illumina sequencing”, and cite the paper describing how the genotypes were obtained (Davies et al., Nature Genetics 2016)*

6.	Should there be citations in structured abstract?

*We have removed all citations from the abstract*

7.	Figure 2 - would be nice if you could label the phenotypes in small type We have added this information in Figure 2 of our revised manuscript.

8.	Color coding of the phenotype categories in Fig 3 does not really add much so I'm not sure it is necessary

*The color coding helps check visually that nearby significant loci are not for the same nor related phenotypes, which supports the sentence “There was no overlap between significant DGE and IGE loci for the same phenotype, or even for related phenotypes”. Therefore, we wish to retain the color unless this reviewer strongly disagrees.*

9.	Supplementary note - please write out iid at first use

*We have made this change*

0.	P. 25 - capitalize "parker"

*We have capitalized the first letter of Parker*

1.	Table captions and/or footnotes/abbreviations would be helpful for the Supplemental Tables. For example it is unclear what things like Weight.Diss, Weight.Hypo, etc. mean - is this weight at dissection? Weight of hypothalamus? hypoxia? What are NB_slices? Many of the phenotypes and abbreviations are intuitive but not all. The Weights are listed at bottom but not intuitive when viewed as covariates at the top of the Table. Is the phenotype "NA" in Row 33 a mistake?

*In our revised manuscript we have added two additional columns in each of Supplementary Tables 3 and 4 (significant IGE and DGE loci) to provide further information on the phenotypes. We have corrected the NA to indicate Osteopetrosis.*

12.	Table S2: normalize font size. Please give mouse genome ref build. How were candidate genes at locus selected -- by some distance or linkage bounds? Is this the 1.5 Mb mentioned in the text uniformly applied?

*We have added the genome assembly (mm10) in Supplementary Tables 3 and 4 of our revised manuscript.*

*We describe, in the Methods section, how putative causal genes were identified: “At each significantly associated locus we defined a 1.5Mb window centred on the lead variant corresponding, in this sample, to the 95% confidence interval for the association(33). We identified all the variants that segregate in this window based on the full set of 7M variants and reran igeGWAS and dgeGWAS locally using all the variants at the locus. We defined “candidate” genes as those genes that either overlapped the associated plateau or were located in direct proximity, and whose MGI symbol does not start by ‘Gm’, ‘Rik’, ‘Mir’, ‘Fam’, or ’Tmem’ in order to focus on genes with known function and generate more tractable hypotheses on the pathways of social effects.” In addition, with our revised manuscript we provide the locusZoom plots for all 24 significant IGE loci reported in Supplementary Table 3 (locusZooms_SupplTable3.zip)*

13.	Table S3: as in Table S2 similar comments

*Done*

14.	Table S4: label column A as 'Mouse ID'. It is unclear what the abbreviations in Row 4 indicate - please add to sub-caption/footnote. Mice with "NA" - does this indicate that mice were deceased and not carried forward in the study, or otherwise incapacitated/unable to be tested? If so, please indicate (some mice are NA for all cage conditions). I think it is commendable that the authors have provided this level of detail and data availability to enable future researchers to either reconstruct analyses or attempt additional analyses.

*The cage information, which used to be in Supplementary Table 4, is now in Supplementary Table 1.*

*We have added, in our revised table, “Mouse ID” and spelled out the abbreviations in the titles of the columns.*

*NA in the cage information can point to a mouse that died, changed cage or, more likely, whose cage stopped being recorded at some point (sometimes at the first time point, in which case the mouse has NA at all time points). From the time t a mouse died or the cage of a mouse stopped being recorded, the phenotypes collected at t’ >= t for the mouse and all its previous cage mates were turned to NA, hence effectively excluding these mice from the corresponding DGE and IGE analyses. We have added this information in the legend of Supplementary Table 1 of our revised manuscript.*

Comments from Reviewer #2

Baud et al describe the genetic architecture of direct (DGE) and social genetic (SGE) effects for 170 traits measured in outbred mice. They report 24 and 120 genome-wide associated loci for SGE and DGE, respectively. The paper is well written and the general area is of interest but the manuscript has two main issues:

1) there is not attempt to replicate the findings or understand their functional significance. Without replication it is impossible to assess to what degree the reported loci are false positives.

*We agree with the Reviewer that replication or validation of SGE (now denoted IGE in the revised manuscript) would be a major advance for igeGWAS. Whereas replication in human genetics typically means repeating the same analysis in an independent cohort, another type of replication is possible in model organisms, because implicated genes can be mutated or deleted to assess whether or not they influence the phenotype of interest.*

*Concerning the replication in a second cohort, there exists unfortunately no appropriate cohort for replicating our analyses. Indeed, there is only one other CFW cohort, from Parker et al. Nature Genetics 2016, with the genotype and phenotype data necessary to carry out GWAS and only one of the phenotypes for which we report a significant IGE locus in this manuscript, namely osteopetrosis, was also measured in the Parker cohort. Therefore, quantifying the replication rate for IGE loci using the Parker cohort is not feasible. In addition, Zou et al. bioRxiv 2021 ( https://doi.org/10.1101/2021.02.05.429998) demonstrated that the relatively low replication rate for DGE loci between the cohort used in our study and the Parker cohort was due not only to the winner’s curse but also to experimental heterogeneity between the two studies, likely born from the use of slightly different protocols by the two teams in these studies. For all these reasons we feel that the Parker cohort is a poor candidate to try to replicate the IGE locus for osteopetrosis. Altogether, therefore, replication using an independent cohort was not a viable strategy for our study.*

*As an alternative, in our revised manuscript, we have considered the second type of replication mentioned above. Specifically, we report on a follow up experiment to evaluate a putative causal gene, Epha4, which we identified in CFW mice for IGE on both stress-coping strategy (significant) and wound healing (suggestive). In this new experiment we used Epha4 knockout mice as cage mates and inbred FVB mice as focal mice. The FVB background was chosen because it is the inbred genetic background that is closest to that of CFW mice (Nicod et al. Nature Genetics 2016). These novel experimental data support the role of Epha4 in giving rise to IGE on stress-coping behaviour and wound healing in male mice (P values 0.054 and 0.038 respectively, Figure 5).*

*Although this experiment does not address the replication rate of the IGE loci we identified in igeGWAS, it supports that our analyses yield genuine IGE associations and, in addition, it validates the role of a specific gene in giving rise to IGE.*

*We present these experimental results in the Results section entitled “Evaluating the role of Epha4 and Dlgap1 in IGE using knockout models” of our revised manuscript.*

2) the authors, who are worried about having related mice in the same cage, end up using a very ad-hoc approach to avoid removing 435 mice. Whilst I can understand that they want to make a compromise between removing the effect of relatedness and losing statistical power. The truth is that the reader ends up not knowing whether the effect would still persist if the related individuals were removed. In a way, that the genotype of a group of individuals explain variation of their cousins is not that surprising, and keeping only a few cousins could lead to that effect. The authors could perhaps try to see if the effects hold after removing the 435 mice.

*There is a misunderstanding here: we do not worry that spurious IGE might arise from confounding between the genotypes of the focal mice and the genotypes at the same variant of their (possibly related) cage mates. This is indeed unlikely because, firstly, suspected close relatives (“close pairs”) were largely spread across cages rather than sharing a cage and, secondly, because DGE and IGE were modelled jointly when estimating the aggregate contribution of IGE and DGE of the tested locus were accounted for in the null model for igeGWAS. Accounting for DGE of the tested locus in igeGWAS is key to avoiding confounding *even when strictly unrelated mice are considered*, as we demonstrate in the Supplementary Note.*

*What we are trying to avoid, by excluding 57 mice suspected to be close relatives, is confounding from environmental effects shared by close relatives, which may be present and could bias both DGE and IGE analyses. Close relatives were not supposed to be included in the sample but the long tail of high GRM values suggested that, perhaps, close relatives had accidentally been included (the exact genetic relationship values for sibglings, etc. are unknown as the CFW colony is of limited size and breeding occurs between somewhat related mice).*

*Nevertheless, we agree that a conservative analysis, whereby suspected close relatives are discarded more aggressively, can provide additional confidence. Following the suggestion made by this Reviewer we repeated all analyses after excluding all 435 mice suspected to be closely related to at least one mouse in the study. The results are presented in several figures and tables below. We have not included them in our Supplementary Information but could do so if the Reviewer thought this was necessary.*

*·	The figure below shows that the aggregate contributions of DGE (panel on the left) and IGE (panel on the right) reported in the original manuscript are not biased by the exclusion of 57 mice only:*

*The tables below shows that excluding 57 or 435 mice yields similar results with regard to the correlation between DGE and IGE. Indeed, phenotypes with significant P values for the test “ρ different from zero” (table on the left) or “ρ different from plus/minus one” (table on the right) in our manuscript, which are highlighted in yellow, also tend to have the smallest P values when all 435 mice are excluded. All 28 phenotypes for which ρ was estimated are shown below.*

*·The manhattans plots for igeGWAS presented in manhattan_plots_57or435_mice.pdf show that all the significant IGE loci reported in Supplementary Table 3 of our revised manuscript (highlighted in bright colours in each Manhattan plot) remain strongly associated after excluding all 435 mice, although significance levels are generally lower as expected from the smaller sample size.*

Some of the methods are a bit unclear, for instance, how the genotypes of the two mates in a cage are combined into a single component of variance.

*The social breeding values of the two cage mates are summed (! " in Equation 0, Methods section). We have tried to clarify this in the revised paper: “For igeGWAS, we estimated the “social genotype” of a mouse at a variant as the sum of the reference allele dosages across its two cage mates at the variant(30, 39), and tested for association between this social genotype and the phenotype of interest.” (Results section, L164 – 167).*

On a minor, one of the formula got very weird (page 21). *We have corrected this formula*

**Second round of review**

**Reviewer 1**

A blast from the past so to speak. I re-familiarized myself with the authors' work and their detailed responses to reviewer comments. Overall I think the authors have adequately responded to comments, clarified the manuscript at points and taken an important step in testing the knockout models. My remaining comments are minor in nature:

1) In regards to Epha4 and the role in wound healing there is some interesting literature (some new during this revision period and some old) that may be worth citing in your discussion. Particularly some of this work implicates Epha4 for roles in regulation of endothelial function/vascular remodeling, and several works (take your pick) including early work by Skip Brass in the 2000s showing a role for Epha4 in platelet function. These studies likely serve to underline a very plausible role for Epha4 in wound healing possibly through multiple mechanisms. Some references to consider:

https://pubmed.ncbi.nlm.nih.gov/25370417/

https://pubmed.ncbi.nlm.nih.gov/30817459/

https://pubmed.ncbi.nlm.nih.gov/31689239/

2) The authors have changed to IGE throughout the manuscript. One place missed is Figure 3 (top panel) where SGE was kept, so this should be changed

3) In Figure 4C panel there appears to be a problem with text over-writing the Epha1 gene label which should be corrected

**Reviewer 2**

I'd like to congratulate the authors for the nice work. They have addressed my main concerned about replication. I thought the experiments on Epha4 and Dlgap1 were very clever.

Their conclusions related to IGE not being genetically correlated with IGE are consistent with a recent report in humans (https://doi.org/10.1038/s41562-020-00991-9) and this substantiates both works. They might consider citing this paper in the current MS.

My only concern is the interpretation of the results related to Epha4. I might be wrong but the main experiment is not significant (pval=0.52 and 0.4 for FST immobility and wound healing), the sex stratified analyses seem to suggest that there is an effect in males but not females for both traits. But is this really significant? Even assuming only 2 independent hypotheses tested and assuming 1 trait has been tested, a p=0.038 is (0.05/2=0.025) is not really significant. Now that the authors have an estimated effect size, is there any chance of increasing N to have sufficient power to detect the effect with a well powered study?

**Authors Response**

**Point-by-point responses to the reviewers’ comments:**

Reviewer #1: A blast from the past so to speak. I re-familiarized myself with the authors' work and their detailed responses to reviewer comments. Overall I think the authors have adequately responded to comments, clarified the manuscript at points and taken an important step in testing the knockout models. My remaining comments are minor in nature:

1) In regards to Epha4 and the role in wound healing there is some interesting literature (some new during this revision period and some old) that may be worth citing in your discussion. Particularly some of this work implicates Epha4 for roles in regulation of endothelial function/vascular remodeling, and several works (take your pick) including early work by Skip Brass in the 2000s showing a role for Epha4 in platelet function. These studies likely serve to underline a very plausible role for Epha4 in wound healing possibly through multiple mechanisms. Some references to consider:

https://pubmed.ncbi.nlm.nih.gov/25370417/

https://pubmed.ncbi.nlm.nih.gov/30817459/

https://pubmed.ncbi.nlm.nih.gov/31689239/

*Response:*

*We reviewed the work indicated above looking for a mechanism that could explain how expression of Epha4 in cage mates influences wound healing in focal mice. We found that Epha4 is expressed in platelets and that platelets are present in saliva. Given the well-known beneficial role of saliva and platelets for wound healing we hypothesized that expression of Epha4 in the cage mates’ platelets can affect healing of the focal mice after their wounds are licked by their cage mates. We have included this hypothesis in our discussion of how IGE genes can help generate testable hypotheses as to the mechanisms of IGE.*

2) The authors have changed to IGE throughout the manuscript. One place missed is Figure 3 (top panel) where SGE was kept, so this should be changed

*Response:*

*We have changed SGE to IGE in Figure 3.*

3) In Figure 4C panel there appears to be a problem with text over-writing the Epha1 gene label which should be corrected

*This over-writing in the figure in the Manuscript PDF is not present in the original figure, which we submitted as an independent file. So we think that will not be an issue for publication.*

Reviewer #2: I'd like to congratulate the authors for the nice work. They have addressed my main concerned about replication. I thought the experiments on Epha4 and Dlgap1 were very clever.

Their conclusions related to IGE not being genetically correlated with IGE are consistent with a recent report in humans (https://doi.org/10.1038/s41562-020-00991-9) and this substantiates both works. They might consider citing this paper in the current MS.

*Response:*

*We have indicated in our discussion that “a similar conclusion has been reached in a study of spouses in the UK BioBank(26).”, reference included (reference also included in the introduction on peer genetic effects).*

My only concern is the interpretation of the results related to Epha4. I might be wrong but the main experiment is not significant (pval=0.52 and 0.4 for FST immobility and wound healing), the sex stratified analyses seem to suggest that there is an effect in males but not females for both traits. But is this really significant? Even assuming only 2 independent hypotheses tested and assuming 1 trait has been tested, a p=0.038 is (0.05/2=0.025) is not really significant. Now that the authors have an estimated effect size, is there any chance of increasing N to have sufficient power to detect the effect with a well powered study?

*Response:*

*The P values from the sex-stratified analysis are, indeed, suggestive rather than significant. Together with other lines of evidence, however, they contribute to making a strong case for a role of Epha4 in mediating IGE on stress coping strategy and wound healing. These lines of evidence are the following:*

*1) First, there is a significant association between variants in the vicinity of Epha4 and stress-coping strategy in the igeGWAS carried out in CFW mice. The Epha4 locus is in fact in the top half of the list of IGE loci in terms of significance.*

*2) Secondly, Epha4 is the only putative causal gene at the locus, as defined in the Methods section. We have also added to our revised manuscript that “Epha4 is in a gene desert and the only protein-coding gene in a chromatin topological domain(47, 48), meaning the causal variant(s) at the Epha4 locus likely affect the expression of Epha4 and not that of a neighbouring gene.”*

*3) Turning now to the experiment with the Epha4 KO mice now, model selection using the Akaike Information Criterion favours a model with sex of cage mate, IGE and an interaction between them for both phenotypes, indicating that there are IGE from Epha4 on both phenotypes, but that this effect differs between males and females*

*4) As expected from the model selection analysis, ANOVA yields suggestive evidence of IGE on both phenotypes in males (P = 0.053 and 0.038) but not in females.*

*In the discussion of our revised manuscript we have recapitulated these lines of evidence and qualified the ANOVA results of “suggestive” to not overstate our findings.*

*We are unfortunately unable to test additional mice at this point as we are in the process of transferring the colony from the University of California San Diego to the Centre for Genomic Regulation in Barcelona, where we will carry out further work on IGE from Epha4.*
